# A molecular roadmap to the plant immune system

**DOI:** 10.1074/jbc.REV120.010852

**Published:** 2020-08-17

**Authors:** Adam R. Bentham, Juan Carlos De la Concepcion, Nitika Mukhi, Rafał Zdrzałek, Markus Draeger, Danylo Gorenkin, Richard K. Hughes, Mark J. Banfield

**Affiliations:** Department of Biological Chemistry, John Innes Centre, Norwich, United Kingdom

**Keywords:** plant immunity, cell-surface immunity, intracellular immunity, resistance engineering, effectors, nucleotide-binding leucine-rich repeat receptors (NLRs), receptor-like kinases (RLKs), receptor-like proteins (RLPs), plant defense, plant biochemistry, Nod-like receptor (NLR), cell surface receptor, cellular immune response

## Abstract

Plant diseases caused by pathogens and pests are a constant threat to global food security. Direct crop losses and the measures used to control disease (*e.g.* application of pesticides) have significant agricultural, economic, and societal impacts. Therefore, it is essential that we understand the molecular mechanisms of the plant immune system, a system that allows plants to resist attack from a wide variety of organisms ranging from viruses to insects. Here, we provide a roadmap to plant immunity, with a focus on cell-surface and intracellular immune receptors. We describe how these receptors perceive signatures of pathogens and pests and initiate immune pathways. We merge existing concepts with new insights gained from recent breakthroughs on the structure and function of plant immune receptors, which have generated a shift in our understanding of cell-surface and intracellular immunity and the interplay between the two. Finally, we use our current understanding of plant immunity as context to discuss the potential of engineering the plant immune system with the aim of bolstering plant defenses against disease.

Plants suffer from disease. Their ability to respond to infection by microbial pathogens and pests is essential for survival. In agriculture, plant disease leads to loss in crop yield and can have devastating effects on both subsistence/small-holder and industrialized farming (1–3), with subsequent impact on food supply chains and prices. Plant diseases have also shaped our world, with perhaps the best-known example being the Irish potato famine in the mid-1800s, where potato late blight disease (caused by the filamentous plant pathogen *Phytophthora infestans*) contributed to mass emigration from Ireland (4).

As a rich source of nutrients, plants are the target of microbial pathogens and pests, including viruses, bacteria, filamentous pathogens (fungi and oomycetes), nematodes, and insects to complete their life cycle (5–8). Estimates of the impact of pre-harvest yield loss in crops due to disease vary, but at least 30% of global agricultural production is claimed annually (1). This can increase to 100% in localized outbreaks and represents a major contributor to food insecurity. In agriculture, plant diseases are largely controlled by chemicals, but this is unsustainable in the long-term due to environmental concerns and the necessity to rethink agricultural practices more generally in light of the climate emergency. Genetic forms of disease resistance offer the potential for environmentally friendly, low-input, sustainable agriculture (9). Over the last 25 years, remarkable progress has been made in our understanding of the molecular basis of plant disease resistance mechanisms. Plant immune receptors, encoded by resistance or “R” genes have been cloned and characterized and shown to be the genetic basis of disease resistance phenotypes used by plant breeders for >100 years. Recent studies have extended our knowledge to reveal our first insights into the structural basis of plant immune receptor function (10–19).

The immune system of plants shares similarities with the innate immune system of animals (20–22). But as plants lack an adaptive immune system, they rely solely on innate immunity to recognize microbial pathogens and pests. Conceptually, plant immunity can be divided into cell-surface and intracellular immunity (23). A full list of the structurally characterized immune receptors and associated ligands can be found in Table 1. Cell-surface immune receptors detect common signatures of pathogens or pests outside the host cell via extracellular domains (ECDs) and initiate cellular responses to resist infection via their intracellular kinase domains (KDs) (39). A subset of cell-surface immune receptors sense damaged “self” as a surrogate for the presence of pathogens or pests (15). Intracellular immune receptors detect signatures of adapted pathogens or pests (40). Typically, these signatures are translocated proteins known as “effectors,” which are delivered inside cells to modulate host physiology to promote colonization and proliferation (41, 42) (Fig. 1). Activation of intracellular immunity is generally considered a more robust response and can be associated with localized cell death that constrains the spread of infection. Although often presented as distinct signaling pathways, insights into how cell-surface and intracellular immune pathways in plants overlap and work synergistically to resist infection have recently begun to emerge (43, 44).

**Figure 1.**
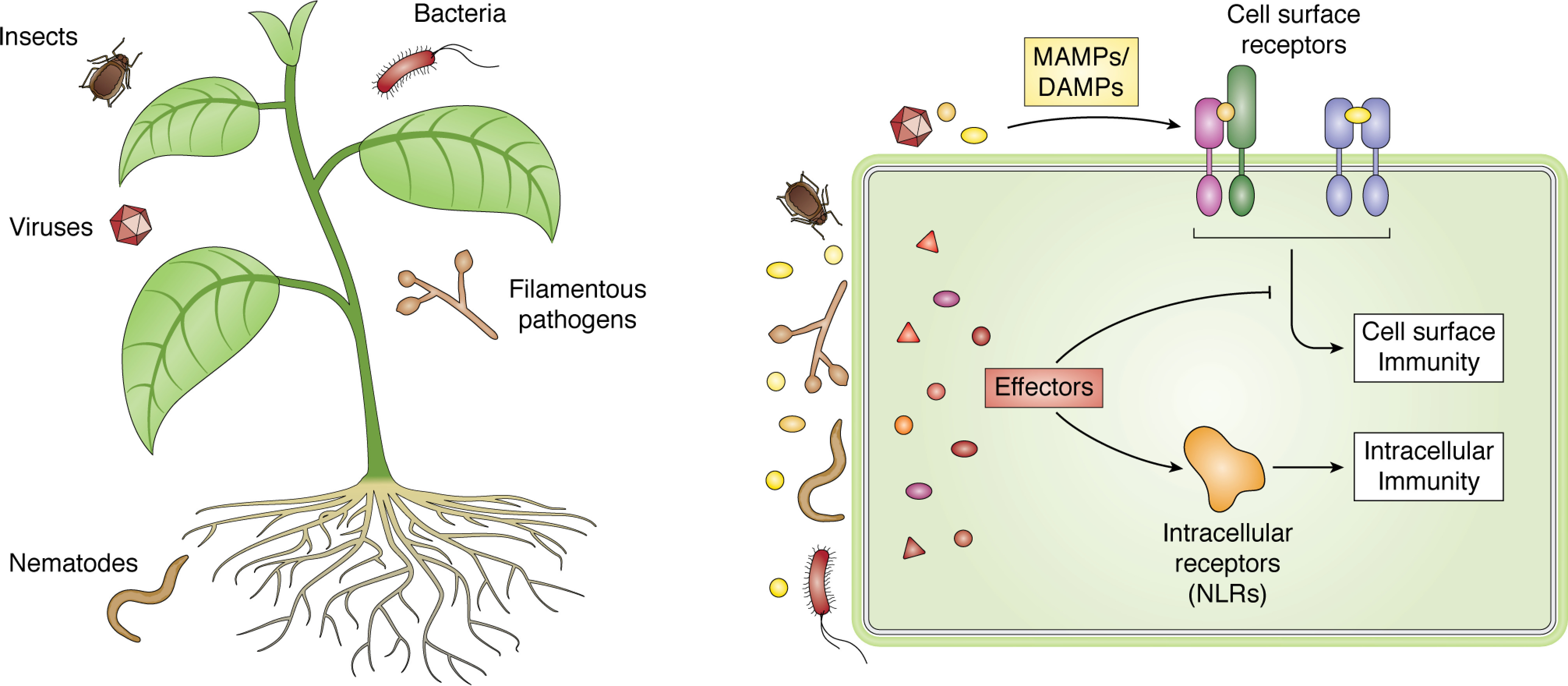
**Plant immunity at a glance.** *Left*, plants are the target of a variety of pathogens and pests that cause disease, via both their above-ground and underground structures. *Right*, pathogens/pests shed MAMPs or generate DAMPs that can be received by receptors to initiate cell-surface immunity. Pathogens/pests can deliver effectors to the outside (not shown here for simplicity) or inside of cells, where they can act on host systems to their benefit, including the suppression of signaling pathways downstream of cell-surface receptors. Effectors or their activities can be sensed by intracellular immune receptors (NLRs) to initiate intracellular immunity.

**Table 1:** **Structures of plant immune receptors or their domains covered in this review**

Receptor	Type: Cell-surface	Plant host	Ligand	Ligand type	Co-receptor	PDB code	References
FLS2	LRR-RLK	*Arabidopsis thaliana*	flg22	MAMP	BAK1	4MN8	12
PEPR1	LRR-RLK	*A. thaliana*	Atpep	DAMP	BAK1	5GR8	13
CERK1	LysM-RLK	*A. thaliana*	PGN	MAMP	LYM3/1	4EBY	10
SOBIR1	LRR-RLK	*A. thaliana*	NA*^a^*	NA	LRR-RLP, BAK1	6R1H	19
BIR3	Pseudokinase	*A. thaliana*	NA	NA	BRI1/S.E.RK1	6FG8	24
BIK1	RLCK	*A. thaliana*	NA	NA	BAK1, FLS2	5TOS	25
CEBiP	LysM-RLP	*Oryza sativa*	Chitin	MAMP	OsCERK1	5JCD, 5JCE	26
**Receptor**	**Type: Intracellular**	**Plant host**	**Ligand(s)**	**Ligand type**	**Co-receptor**	**PDB code**	**References**
MLA10 CC	CC-NLR	*Hordeum vulgare*	NA	NA	NA	3QFL, 5T1Y	27, 28
Pikp-1 HMA	CC-NLR	*O. sativa*	AVR-PikD, AVR-PikE, AVR-PikA, AVR-Pia	MAX effector	Pikp-2	5A6W, 5A6P, 6G11, 6G10, 6Q76	11, 29, 30
Pikm-1 HMA	CC-NLR	*O. sativa*	AVR-PikE, AVR-PikA, AVR-PikD	MAX effector	Pikm-2	6FUB, 6FUD, 6FU9	29
Pia HMA	CC-NLR	*O. sativa*	Avr1-CO39	MAX effector	RGA4	5ZNG, 5ZNE	31
RRS1 WRKY	TIR-NLR	*A. thaliana*	PopP2	T3SE	RPS4	5W3X	17
ZAR1	CC-NLR	*A. thaliana*	Avr-AC	T3SE	RKS1	6J5T, 6J6I, 6J5W, 6J5V	14, 15
RPS4 TIR	TIR-NLR	*A. thaliana*	NA	NA	RRS1	4C6T, 4C6R,	16
RRS1 TIR	TIR-NLR	*A. thaliana*	NA	NA	RPS4	4C6T,4C6S	16
SNC1 TIR	TIR-NLR	*A. thaliana*	NA	NA	NA	5TEC	32
SNC1 TIR	TIR-NLR	*A. thaliana*	NA	NA	NA	5H3C	33
Sr33 CC	CC-NLR	*Aegilops tauschii*	NA	NA	NA	2NCG	28
RPP1 TIR	TIR-NLR	*A. thaliana*	NA	NA	NA	5TEB	32
NRC1 NB-ARC	TIR-NLR	*Solanum lycopersicum*	NA	NA	NA	6S2P	34
RUN1 TIR	TIR-NLR	*Vitis rotundifolia*	NA	NA	NA	60OW	35
Rx CC	CC-NLR	*Solanum tuberosum*	NA	NA	RanGAP2	4M70	18
RPV1 TIR	TIR-NLR	*Vitis rotundifolia*	NA	NA	NA	5KU7	36
L6 TIR	TIR-NLR	*Linum usitatissimumm*	NA	NA	NA	3OZI	37
Pto	Kinase	*Solanum pimpinellifolium*	AvrPtoB	T3SE	Prf	3HGK	38

*^a^*NA, not applicable.

There are many excellent reviews covering plant immunity and its subversion by microbial pathogens and pests published over the last ∼15 years (21, 39, 45–53). Here, as part of this JBC “Plants in the Real World” thematic series, we provide an up-to-date overview of the general concepts of plant disease resistance mechanisms, with a focus on plant immune receptor function at the molecular level. We detail how these receptors perceive pathogen signatures at the cell surface and inside host cells and how this perception is translated into an immune response. This review summarizes the general concepts of plant immunity before providing in-depth analyses of the more recent breakthroughs that have greatly expanded our understanding of plant immune receptor function. Finally, in the context of current knowledge, we discuss how plant immune receptors could be engineered to deliver novel disease resistance properties to benefit global food security.

## Effectors: Master manipulators of plant cells to promote infection

To best understand the interplay between the pathogens/pests and the plant immune system, we must first understand effectors and their role in promoting virulence. In the broadest definition, effectors are molecules used by a diverse array of organisms (including microbes, plants, and animals) to modulate the activity of another organism. In this review, we use the term “effectors” to define protein molecules secreted by microbial pathogens and pests to promote colonization of their plant hosts (53). These effectors can be delivered to the extracellular space or deployed to the inside of host cells.

Effector genes exist as large repertoires within pathogen genomes. They are among the most rapidly evolving genes in plant pathogens and can display high rates of nonsynonymous over synonymous mutations (54, 55). Selection for evasion of perception by the plant immune system is a major driver for adaptation, along with selection for favorable alleles that give a fitness benefit (56). Due to their sequence diversity, it is frequently challenging to identify effectors in pathogen/pest genomes or proteomes, although many bioinformatic tools exist to establish putative effector catalogues (57). Functional annotation of effectors is equally challenging. Whereas some effectors are enzymes, whose putative activity can be identified from sequence or structural analysis, many do not show sequence or structural homologies to help define function (49, 58). This often necessitates significant investment in research of a single protein to establish the molecular basis of activity (42). Such research will frequently address the host cell target of an effector, as this is often key to understanding its role in virulence. Some effectors converge on “hubs,” key components of host cell pathways, to modulate their function (59, 60). Such pathways include suppression of defense responses (Fig. 1) and redirection of host metabolism.

Effectors are also an Achilles' heel for the pathogen/pest. As signatures of non-self, they can be perceived by plant immune receptors at both the cell surface and inside cells. Intracellular perception of effectors or their activities is mediated and transduced by NLRs, as described elsewhere in this review.

## Cell-surface immunity

Two major components of cell-surface immunity in plants are membrane-localized receptor-like kinases (RLKs) and receptor-like proteins (RLPs) that detect signatures of non-self as signs of infection (45). RLKs/RLPs also have other roles in plants, regulating self-incompatibility, growth and development, reproduction, response to abiotic stress, and symbiosis (45, 61–63). Also known as pattern recognition receptors (PRRs), cell-surface immune receptors monitor the extracellular environment for pathogen/pest invasion patterns (ligands known as MAMPs (microbial-associated molecular patterns) or DAMPs (damage-associated molecular patterns)) (64, 65). Frequently, ligand-sensing cell-surface receptors require co-receptors to transduce perception of non-self into a response (66, 67). Although proteinaceous receptors represent the major players in cell-surface immunity of plants, recent studies have highlighted an emerging role of membrane lipids in sensing infection (50).

Irrespective of their origin, invasion patterns recognized by cell-surface immune receptors tend to be evolutionarily constrained ligands derived from components essential to the fitness of the pathogen/pest. These essential components range from cell wall constituents or subunits of bacterial flagellin, to molecules secreted into the apoplast, to secreted proteins intended for the host cytosol (39). These specific ligands are perceived by cell-surface receptors at nanomolar concentrations and initiate signaling cascades, including production of reactive oxygen species (ROS), cytosolic Ca^2+^ bursts, activation of MAPKs, and changes in expression of various defense-related genes (64, 67, 68). Generally, cell-surface immune responses are considered less volatile when compared with intracellular immunity and do not result in host cell death to restrict infection. However, they constitute an effective host strategy against infection, leading to broad-spectrum resistance (69). This review focuses on the mechanisms of immune activation rather than the downstream effects of extracellular and intracellular immunity; for readers interested in the physiological effects of immune activation, we recommend relevant reviews (70–72).

Signaling cascades downstream of cell-surface immune receptors are major targets of pathogen/pest effector proteins, which interfere with these processes to benefit infection. It is also worth noting that many MAMPs are shared between pathogens and mutualistic microbes (62, 73), and as such it is important to understand how plants use extracellular immune receptors to distinguish between pathogens/pests and mutualists. In this review, we cover MAMP recognition from a pathogen/pest-detection perspective and would direct readers interested in plant-mutualist interaction to relevant reviews (62, 73).

### Structural and functional diversity of ligand recognition by cell-surface receptors

**Figure 2.**
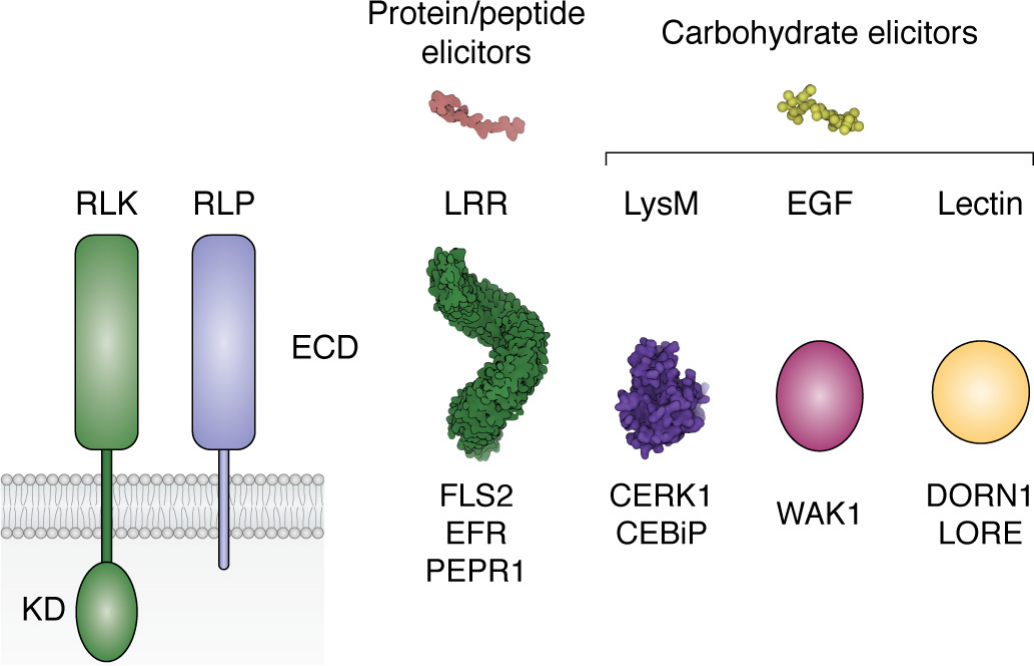
**Diversity of cell-surface immune receptors.** A *schematic representation* depicts the domain architecture of different classes of plant RLKs/RLPs. *Surface representations* are shown for those ECDs for which crystal structures are available. *LRR*, crystal structure of the ECD of *Arabidopsis* RLK FLS2, PDB entry 4MNA (*green*); *LysM*, crystal structure of the ECD of *Arabidopsis* RLK–CERK1, PDB entry 4EBY (*purple*).

RLKs contain a variable extracellular domain that mediates ligand recognition, a single-pass transmembrane domain, and an intracellular KD that transduces the signal to downstream immune components (74) (Fig. 2). Most plant RLKs identified belong to the family of non-RD kinases (defined by the absence of conserved arginine in the catalytic loop) and often associate in dynamic complexes with membrane-bound RLKs that are functional RD kinases (such as BAK1 (BRASSINOSTEROID-INSENSITIVE 1–associated receptor kinase 1) and SERKs), which operate as co-receptors for perception to initiate immune signaling (75–77). Whereas RLPs exhibit a similar overall structure to RLKs, they only contain a short intracellular tail, lacking a kinase domain, and require a partner co-receptor to signal (63, 78).

**Figure 3.**
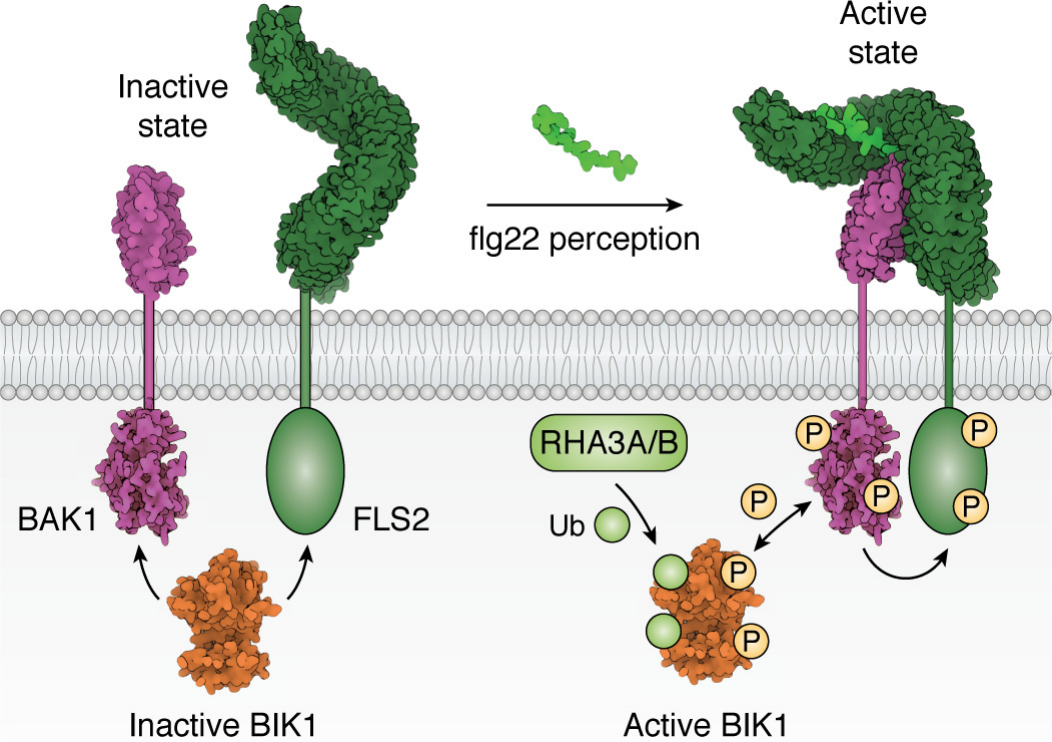
**A mechanistic view of flg22 sensing by FLS2.** flg22 (*light green*) stabilizes the heterodimerization of FLS2 (*dark green*, PDB entries 4NMA and 4NM8) with BAK1 (*purple*, PDB entries 3ULZ and 4NM8) (82, 85, 86). Ligand perception leads to activation and phosphorylation of BIK1 (*orange*, PDB entry 5TOS) by BAK1 (87, 88). Following phosphorylation, BIK1 is monoubiquitinated (*Ub*) by the E3 ligases RHA3A/B. Monoubiquitinated BIK1 is then released from the FLS2–BAK1 complex and initiates ROS production and Ca^2+^ signaling through phosphorylation of plasma membrane–localized NADPH oxidases and cyclic nucleotide–gated channels (89). The *bidirectional arrow* indicates that both BIK1 and BAK1 can *trans*-phosphorylate each other.

Based on the type of ECD, RLKs and RLPs can be clustered into distinct subfamilies, including leucine-rich repeat (LRR), lysin motif (LysM), lectin, and epidermal growth factor (EGF) domain–containing receptors (66, 79, 80) (Fig. 2). The type of ECD mainly defines the nature of the ligand perceived by the RLK/RLPs; however, a few anomalies persist. Among the best-characterized cell-surface immune receptors are the *Arabidopsis* LRR-type RLKs, FLS2 (flagellin-sensitive 2) and EFR (elongation factor Tu (EF-Tu) receptor) (81, 82), and the LysM-type RLKs LYK5 (lysin motif receptor kinase 5) and CERK1 (chitin elicitor receptor kinase 1) (83, 84). FLS2 (Fig. 3) and EFR recognize peptide epitopes from the N termini of bacterial flagellin (flg22) and bacterial EF-Tu (elf18), respectively (90), whereas LYK5 and CERK1 bind fungal chitin oligomers (84).

### Recognition of peptide/protein ligands

LRR-RLKs are a large subfamily of cell-surface receptors that preferentially bind peptides or proteins as ligands (91–93). In addition to the *Arabidopsis* FLS2 and EFR, LRR-RLKs from rice and solanaceous plants have been characterized. The rice cell-surface receptor Xa21 binds RaxX21-sY (a sulfated, 21-amino acid synthetic RaxX peptide), a tyrosine-sulfated protein from bacteria (94). Cell-surface receptors from tomato (CORE) and tobacco (*Nb*CSPR) bind to conserved epitopes derived from bacterial cold shock protein (95–97). Likewise, *Arabidopsis* RLP23 binds the epitope nlp-20, a conserved peptide derived from ethylene-inducing peptide 1–like proteins of bacterial and filamentous pathogens (98).

Although not an LRR-RLK, the *Arabidopsis* cell-surface receptor FERONIA (FER) uses a tandem malectin-like ECD to perceive RALF1 (rapid alkalinization factor 1) peptides. RALF peptides are cysteine-rich peptides prevalent in the plant kingdom that regulate many aspects of plant life, such as reproduction, growth, responses to environment, and immunity (99, 100). Intriguingly, some functionally active RALF-like peptides have been characterized from fungal pathogens; however, the role of these RALF-like peptides in pathogenesis is unknown (101). In addition to MAMP ligands, some LRR-RLKs perceive proteinaceous DAMPs, such as Atpeps (plant elicitor peptides) and PIPs (PAMP-induced secreted peptides), respectively (102–105). Like LRR-RLKs, LRR-RLPs can also sense extracellular short peptide ligands; however, they can also sense larger extracellular proteinaceous ligands, such as apoplastic effectors. In tomato, the LRR-RLPs Cf-2/4/9 perceive apoplastic effectors Avr2, Avr4, and Avr9 from *Cladosporium fulvum*, respectively (106–110).

### Recognition of carbohydrate/non-proteinaceous ligands

There are several different classes of receptor that are capable of sensing different carbohydrate ligands. LysM-RLKs/LysM-RLPs perceive carbohydrate MAMPs such as bacterial peptidoglycan (PGN), lipopolysaccharide (LPS), and fungal chitin (10, 83, 84, 111, 112). The ECD of the cell wall-associated kinase family (WAKs) comprise repeated EGF-like domains (113–116) that bind various types of pectins including pathogen/wound-induced short oligogalacturonic acid fragments (OG) as well as cell wall- associated longer pectins (116, 117). Intriguingly, lectin RLKs including structurally distinct lectin receptors - LORE (G-type lectins) and DORN1 (L-type lectins) senses non-carbohydrate ligands like low complexity bacterial metabolites such as bacterial medium-chain 3-hydroxy fatty acid (mc-3-OH-FA) (266) and extracellular ATP (e-ATP- as a DAMP signal) (118, 119) respectively, to trigger immune responses.

### Ligand-induced homo/heterodimerization of cell-surface receptors

Plant cell-surface immune receptors function in complex with co-receptors and intracellular kinases to activate defense (65, 66, 78). The LRR-RLK BAK1 is the best-characterized co-receptor to date (13, 77, 120). BAK1 forms heterocomplexes with peptide-binding immunity-related LRR-RLKs, including FLS2 (Fig. 3), EFR, and PEPR1 (perception of the *Arabidopsis* danger signal peptide), and is required for immune signaling (12, 13, 85, 120, 121). Like BAK1, SOBIR1 (suppressor of Bir 1-1) is a regulatory LRR-RLK that serves as an adaptor for certain LRR-RLPs to trigger defense (122–124). Similar to LRR-RLKs, these RLP/adaptor complexes recruit BAK1 or other SERKs for signal transduction (125–127).

By contrast, the *Arabidopsis* carbohydrate-binding LysM-RLK CERK1 forms chitin-bridged homodimers (10). Homodimeric association has also been reported for the chitin-binding rice LysM-RLP CEBiP (128, 129), but the rice CEBiP can also form heterodimers with rice CERK1 (10, 130). Although oligomerization is important, the precise role of homo- or heterointeractions of LysM-RLK/RLPs in signaling recognition of chitin remains unclear (128).

### RLCKs in downstream defense signaling

Ligand perception by plant cell-surface receptors typically results in homo- or heterodimerization that stimulates *cis*- and/or *trans*-phosphorylation of intracellular kinase domains (128). In turn, the kinase domains of cell-surface immune receptors activate receptor-like cytoplasmic kinases (RLCKs) to transduce immune signals (87, 131, 132).

The *Arabidopsis* RLCKs BIK1 (botrytis-induced kinase 1) and PBL (PBS1-like) proteins are substrates of distinct receptor/BAK1/CERK1 complexes at the cell surface (87, 88, 133). For example, in the absence of ligand, BIK1 interacts with BAK1 and associated cell-surface receptor kinase domains (Fig. 3). On ligand binding, a series of *cis*/*trans*-phosphorylation events promotes BIK1 dissociation from the complex (87, 88). BIK1 then activates various downstream immune signaling pathways, including ROS burst, Ca^2+^ accumulation, and mitogen-activated protein kinase pathways (134–136). Multiple RLCKs have been identified in plants that regulate a ROS burst by phosphorylating distinct sites in RBOHD (respiratory burst oxidase homolog protein D), a membrane-localized NADPH oxidase critical for ROS formation post-MAMP detection (25, 135, 137, 138).

### Regulation of cell-surface immune responses

To prevent inappropriate signaling, the activity of plant cell-surface immune receptors is tightly controlled (139). Plants use various strategies to help maintain cell-surface receptors in an inactive state in the absence of ligand binding, including the regulation of phosphorylation status and ubiquitination by E3 ligases (139–142).

Phosphorylation is central to cell-surface immunity signaling cascades and is under tight regulation. Plants use phosphatases to negatively regulate cell-surface receptors to prevent the potentially harmful effects of autoinduction. For example, *Arabidopsis* PP2A (protein phosphatase 2A), a serine/threonine phosphatase, dephosphorylates BAK1/EFR to control defense signaling (143, 144). Similarly, PP2C38 regulates ligand-induced phosphorylation of BIK1, moderating signaling by this key transducer of cell-surface immunity (139). A second strategy to negatively regulate cell-surface immunity is the use of pseudokinases, such as BIR1 and BIR2, that are catalytically inactive but interact with BAK1 in its resting state, preventing the association of LRR-RLKs (145–147). Ligand binding relieves this inhibitory interaction, leading to the formation of activated immune complexes.

Regulation of immunity can also come from controlled degradation through ubiquitination. Two closely related E3-ubiquitin ligases, PUB25 and PUB26, together with both a calcium-dependent protein kinase CPK28 and a heterotrimeric G protein, form a regulatory module and maintain BIK1 homeostasis (148). Similarly, PUB12 and PUB13 polyubiquitinate and mediate degradation of ligand-bound FLS2 (149–151). Intriguingly, a recent study showed that monoubiquitination of BIK1 is necessary for its release from the FLS2/BAK1 complex and immune system activation (89). This demonstrates that a variety of post-translational modifications are important for both positive and negative regulation of cell-surface immune receptors.

In addition to regulating the pool of ligand-bound cell-surface receptors at the plasma membrane, plants also ensure the availability of ligand-free receptors for ongoing pathogen/pest perception. Cell-trafficking components, including SCD1 (DENN domain protein) (152, 153) and ESCRT-I (an endosomal sorting complex required for transport) (154, 155), are involved in delivering these receptors to the cell surface. Finally, it has been proposed that sets of cell-surface receptors may gather at discrete locations on membranes, forming discrete nano- or microdomains (156, 157). These nano-/microdomains are proposed to use similar downstream signaling components; however, different groupings of receptors would lead to different specificity in signal perception, resulting in different responses to stimuli. However, more work is needed to understand the specificity of these nano-/microdomains and how they are clustered into spatially distinct regions of the membrane (156, 157).

### Next steps in understanding cell-surface immunity

Although hundreds of RLKs and RLPs have been identified in many plant species, only a subset have been characterized. The biological significance of the vast majority of these receptors remains elusive, and their underlying mechanism of ligand perception remains poorly understood. Understanding how cell-surface receptors with different ECDs perceive ligands will provide a foundation for engineering broad-spectrum resistance into crop plants (158, 159). Further, our understanding of how RLCKs coordinate their association with different receptors and facilitate distinct signaling outputs is a key challenge for the future. We have yet to understand whether activated cell-surface receptor complexes form higher-order supramolecular signaling units at the plasma membrane, what the molecular identity of these activated immune complex might be, and how they may differ across different ligand/cell-surface receptor pairs. Beyond this, we must endeavor to understand the determinants of specificity of plant cell-surface receptors for MAMPs, as this will provide insight into how plants distinguish the MAMPs of pathogenic microbes from those of the beneficial mutualistic microbes.

## Case study 1: flg22 perception by the FLS2/BAK1 complex—an exemplar of ligand perception by cell-surface receptors

Genetic screens in *Arabidopsis* identified FLS2 as the gene that recognizes a conserved 22-amino acid N-terminal epitope (flg22) of bacterial flagellin to initiate cell-surface immunity (81, 82, 160). FLS2 belongs to the LRR-RLK class XII subfamily and shares homology with TLR5 (Toll-like receptor 5), an LRR-containing receptor that perceives flagellin in mammals (161, 162). Fig. 3 gives a detailed mechanistic view of how flg22 is perceived by FLS2.

Flagellin perception in *Arabidopsis* requires heterodimerization of FLS2 with BAK1 (82, 85, 86). The crystal structure of the ECDs of FLS2 and BAK1, in complex with flg22, revealed the structural basis of flg22 perception (12). The flg22 peptide is bound within the concave surface of the FLS2-ECD, via the leucine-rich repeat subunits LRR3 to LRR16. flg22 interactions with FLS2 are divided into two regions, separated by a kink in the peptide. The N-terminal seven amino acids of flg22 interact with LRRs 3–6, with the C-terminal 14 amino acids binding LRRs 7–16. Numerous hydrogen-bonding, electrostatic, and hydrophobic contacts are formed between flg22 and FLS2. Interactions between the FLS2 and BAK1-ECDs are both receptor- and flg22-mediated, but the peptide acts as a “molecular glue,” stabilizing the heterodimer.

In the absence of flg22, the *Arabidopsis* RLCK BIK1 can associate with the FLS2 and BAK1 kinase domains. Ligand perception leads to activation and phosphorylation of BIK1 by BAK1 (87, 88). Following phosphorylation, BIK1 is monoubiquitinated by the E3 ligases RHA3A/B (RING-H2 FINGER A3A/B). BIK1 has an N-terminal myristoylation motif, and plasma membrane localization of BIK1 is essential for ubiquitination. Monoubiquitinated BIK1 is then released from the FLS2–BAK1 complex and initiates ROS production and Ca^2+^ signaling through phosphorylation of plasma membrane-localized NADPH oxidases and cyclic nucleotide–gated channels (89).

## Intracellular immunity

Intracellular immunity in plants is conferred by nucleotide-binding, leucine-rich repeat receptor proteins (NLRs). NLRs perceive the presence and/or activities of host-translocated effectors, leading to defense responses that may result in programmed cell death to limit the spread of infection (163). Prior to the molecular identification of NLR receptors and effectors, the genetic basis of what we now call intracellular immunity was established as the “gene-for-gene” model. The gene-for-gene model described a requirement for plants to utilize specialized immune receptors encoded by R (resistance) genes to counteract and respond to the effectors encoded by pathogen AVR (avirulence) genes (164).

### NLRs comprise multiple domains with distinct functions

NLRs belong to the AAA+ class of “signal-transducing ATPases with numerous domains” (STAND) ATPases that share a conserved central nucleotide-binding domain across plant, animal, and fungal kingdoms (165). The STAND superfamily includes APAF1, the primary component of the mammalian apoptosome (166), and NLRC4 (NLR family CARD domain–containing protein 4) and NLRP3 (NLR family pyrin domain–containing 3), which are the best-characterized NLRs of the metazoan immune system (20, 167–171).

Classically, plant NLRs comprise a C-terminal LRR domain; a central nucleotide-binding domain known as the NB-ARC (nucleotide-binding domain shared with APAF1, R gene products and CED4 (172); and a variable N-terminal module, which is typically either a TIR (Toll/interleukin-1 receptor/resistance), CC (coiled-coil) domain, or an RPW8 (resistance to powdery mildew 8)-like CC domain (CC-RPW8) (173). Interestingly, LRR domains appear in both cell-surface and intracellular immune receptors and are widely found to be ligand recognition motifs that mediate protein-protein interactions across kingdoms of life. The LRR domain has been implicated in effector recognition for some NLRs, although it is also likely to be important for autoinhibition of the receptor (20, 174, 175). The NB-ARC domain functions as a molecular switch, with effector perception relayed through this domain via nucleotide exchange (ADP for ATP) (48). The N-terminal domains are required for immunity and divide the NLRs into three major classes: TIR-NLRs, CC-NLRs, and RPW8-NLRs (21). Transient expression assays in plants have shown that the N-terminal domains can initiate cell death autonomously and in the absence of an effector (176). Recently, some NLRs have been shown to incorporate additional noncanonical domains into their architecture (177). Known as integrated domains (IDs), these domains can directly interact with effectors (11, 17, 178–180). Intriguingly, many NLR IDs share sequence/structural homology with established virulence-associated host targets of effectors, such as transcription factors or proteins important for cell homeostasis (181). Overall, the individual domains of plant NLRs function together to deliver an effective immune response against pathogen/pests.

### Effector detection: Direct and indirect perception of effectors by plant NLRs

**Figure 4.**
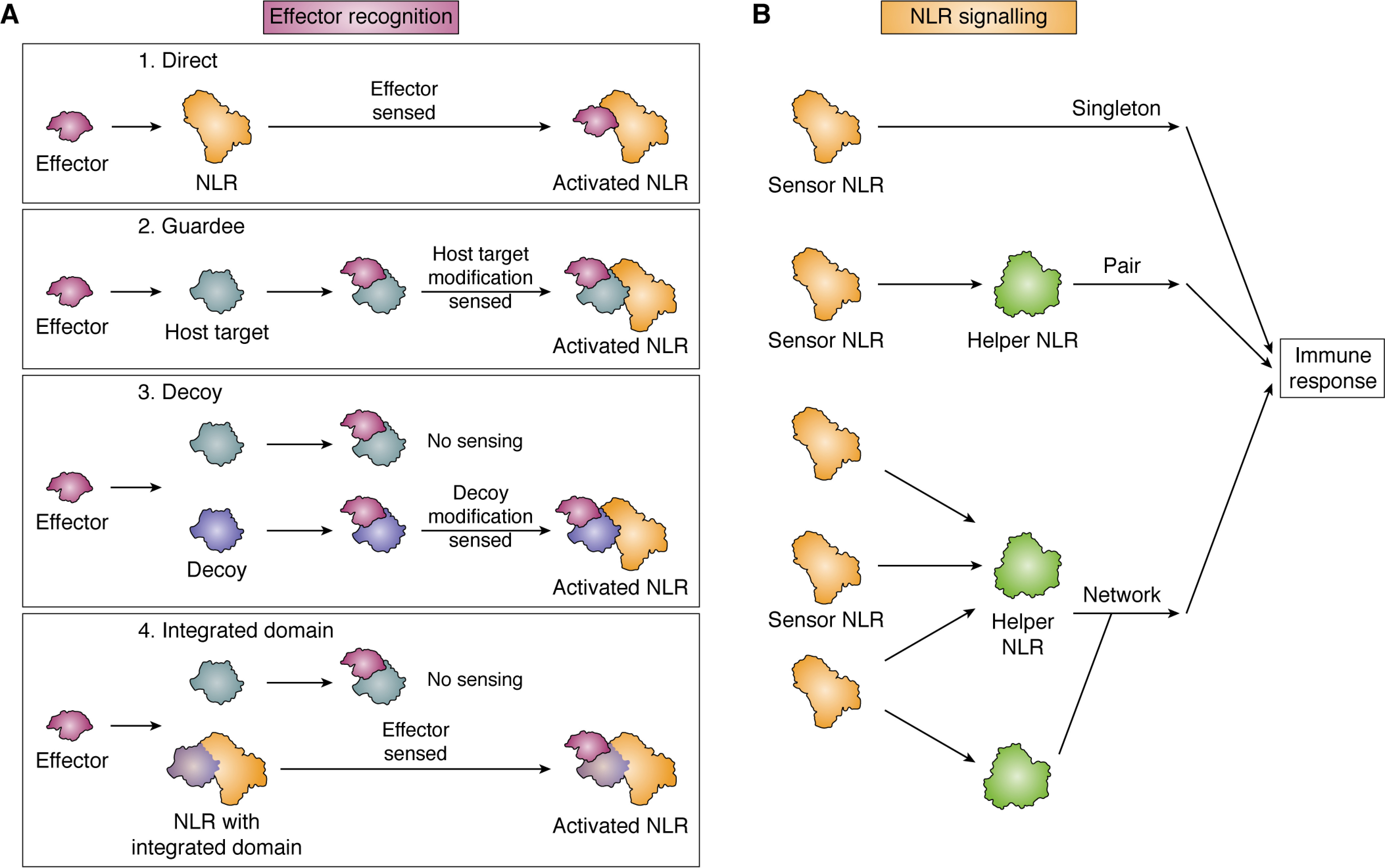
**NLRs perceive effectors via distinct mechanisms and induce immune responses through different mechanisms.** *A*, effector (*purple*) perception induces activation of the NLR (*orange*) via direct binding. NLRs can indirectly perceive and respond to effectors by monitoring modifications of a physiologically relevant host target (*Guardee*, *gray*) or a molecular mimic that likely resulted via gene duplication and is now only involved in immune signaling (*Decoy*, *blue*). NLRs can directly perceive and respond to effectors via NLR integrated domains (*blue*), which likely have their evolutionary origin in ancestral host targets of effectors. *B*, NLR singletons are able to initiate immune responses upon effector perception. Several sensor NLRs require downstream helper NLRs (*green*) to transduce effector perception into immune responses. NLRs can function in pairs or as part of interconnected networks.

**Figure 5.**
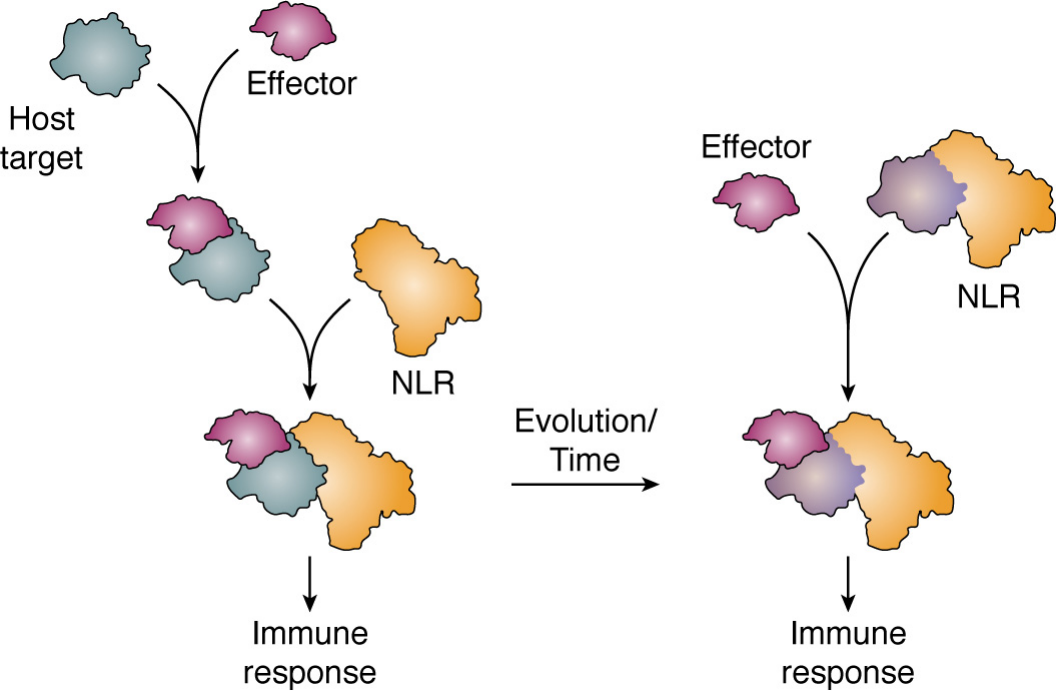
**Incorporation of host targets in NLRs leads to the evolution of NLR with integrated domains.** NLRs (*orange*) can sense changes in host proteins (*gray*) that are targeted by pathogen effector molecules (*purple*) and initiate defense signaling. Over time, some of these host proteins can be found integrated into the NLR core structure (*blue*), acting as the effector recognition domains for the NLR. Binding of an effector to the integrated domain of an NLR leads to initiation of defense responses.

Conceptually, how plant NLRs perceive effectors has been grouped into three overarching models: the direct recognition model (non-ID), indirect recognition model (via guardees or decoys), and the integrated domain recognition model (via integration of effector targets as IDs into the NLR architecture) (Figs. 4*A* and 5).

### Direct recognition

The LRR domain of NLR proteins has been implicated in direct interaction with effectors, as well as having a role in autoinhibition of receptor activity. Best-characterized in flax, this plant shows a variety of resistance phenotypes toward different strains of the flax stem rust pathogen (*Melampsora lini*) expressing different effector alleles (182). In particular, dissection of flax NLRs from the L resistance gene loci (encoding L5, L6, and L7 NLRs among others) and how they perceive alleles of the effector AVRL-567 revealed polymorphisms in the LRR region that underpin specificity (174, 183). Similarly, polymorphisms between the flax NLR variants P and P2 within the LRR domain determine different flax stem rust resistance specificities (184). Although genetic and biochemical evidence for effector perception by LRR domains is established, to date, the structural basis of such interactions has yet to be determined.

### Indirect recognition

NLRs can act as “guards” for host proteins targeted by effectors (known as guardees (181)). Guard/guardee interactions can be divided into two models. In both models, the NLR monitors the biochemical status of the guardee (*e.g.* detecting post-translational modification or cleavage/degradation). In the guard model, the guardee is important for host cell function, whereas in the decoy model, the guardee is a mimic of an effector target but does not have a function outside of immunity.

RIN4 (RPM1 (resistance to *Pseudomonas syringae* pv. *maculicola* 1)-interacting protein 4) is a plasma membrane–localized negative regulator of plant immunity (185). This protein is a classic example of an effector “hub,” a host protein that is targeted by multiple effectors from different pathogens, and as a consequence, it is guarded by multiple NLRs (60). The *Arabidopsis* NLRs RPM1 and RPS2 (resistance to *P. syringae* 2) monitor the biochemical status of RIN4, detecting modifications, such as phosphorylation and degradation, that lead to activation of immunity (185, 186).

In tomato, Pto is a protein kinase that directly interacts with the NLR Prf (187, 188). Pto is a decoy that mimics the intracellular domains of cell-surface immune receptors (187, 188) and acts as a trap for effectors that pathogens have delivered to interfere with receptor signaling. Pto has no known function outside of this bait activity (189). Direct interactions between effectors and Pto lead to oligomerization of Prf and immune activation (188, 189).

### Integrated domain model

The integrated domain model is an evolutionary innovation in plant NLRs where a domain that mimics an effector target is positioned in an NLR architecture, serving as a sensor domain by directly interacting with effectors (Figs. 4*A* and 5). A well-studied example of NLR IDs are the heavy metal–associated (HMA) domains of rice receptor proteins Pik-1 (*Pyricularia oryzae* resistance-k) and the Pia sensor NLR (RGA5; R-gene analog 5), which directly bind effectors of the fungal pathogen *Magnaporthe oryzae* (11, 178). Biochemical, structural, and *in planta* studies have shown how these HMA domains interact with pathogen effectors and demonstrate how different NLR variants perceive different alleles of the effectors (11, 29, 31, 190). Interestingly, a single integrated domain in an NLR can perceive multiple effectors. For example, the WRKY transcription factor–like domain of the *Arabidopsis* NLR RRS1 (resistance to *Ralstonia solanacearum* 1) interacts with two sequence-divergent and structurally divergent effectors (191). One of these effectors adopts a helix-loop-helix fold with an unknown virulence function (AvrRps4 (resistance to *P. syringae* 4); presumed to be a protein-protein interaction module) (17, 192), whereas a second is an acetyltransferase (PopP2) that acetylates both WRKY transcription factors and the RRS1-WRKY (17, 192). The structural basis of interaction between the RRS1-WRKY and PopP2 has been elucidated (17, 192), but the equivalent structure with AvrRps4 remains to be determined. The RRS1-WRKY case demonstrates the versatility of effector perception that integrated domains deliver to NLRs and suggests their utility for receptor engineering.

## Case study 2: Integrated HMA domains—exemplars of integrated domains in NLRs

Many different types of proteins have been found as IDs in plant NLRs, and likely function in direct perception of effectors (193–197). The integrated HMA domains of the sensor NLRs of the rice Pik and Pia pairs are exemplars of IDs and serve as model systems for understanding the principles of effector perception by these domains (11, 29, 31, 178). Fig. 5 illustrates the integration of atypical domains into NLRs to facilitate effector perception.

The integrated HMA domains of Pik-1 and the Pia sensor (also known as RGA5) are likely derived from an expanded family of small plant proteins containing an HMA domain and, sometimes, a C-terminal isoprenylation motif (heavy metal–associated plant proteins (HPPs) or heavy metal-associated isoprenylated proteins (HIPPs) (198)). These proteins may have a role in abiotic stress and detoxification of heavy metals, such as copper or cadmium (198). Additionally, some of these proteins act as susceptibility factors (host targets that can be exploited to assist infection) for diverse pathogens (199–201). This suggests that HPPs/HIPPs may be effector hubs, repeatedly targeted by pathogens as part of infection strategies (59, 60). This provides an evolutionary context for their integration into NLRs as “baits” for triggering immunity (177).

In rice, integrated HMA domains can be found at the C terminus of the sensor NLR of Pia (178) and also between the CC and NB-ARC domain of the sensor NLR Pik-1 (11). Diversity in the location of domain integration implies that these were separate integration events.

The HMA domain of the Pia sensor binds two rice blast effectors, AVR-Pia and AVR1-CO39, whereas the Pik-HMA binds variants of the rice blast effector AVR-Pik (11, 29, 31, 190). Interestingly, these effectors bind to the Pia- and Pik-HMA domains via different interfaces, suggesting that they have independently evolved to target HMA domain–containing proteins, and rice has been able to use both of these interfaces to bait effectors and trigger immunity (31).

As a consequence of arms-race co-evolution with AVR-Pik effector variants (29, 202, 203), the HMA domain is the most variable domain region of the Pik NLRs (204), and the rice Pik receptors also exist as an allelic series with differential recognition specificity for effector variants (202). Biochemical and structural studies of the interaction between different AVR-Pik variants and two allelic HMA domains revealed how subtle changes in the effector/HMA-binding interface underpin variation in recognition specificity (29). Recently, the observation that subtle changes underpin specificity has been used in a proof-of-concept study to show that NLR IDs can be engineered to expand their recognition capacity to allelic effectors (205).

### NLR activation

A general principle of NLR biology is that perception of effectors leads to conformational changes in the receptor. These changes can include domain rearrangements and oligomerization. Whereas the details depend on the mode of effector perception, nucleotide exchange (ADP for ATP) in the NB-ARC domain of NLRs is a factor for activation. Numerous studies have shown the importance of conserved sequences such as the “P-loop” and “MHD-like” motifs (a consensus sequence (methionine-histidine-aspartate) at the C terminus of ARC2) in nucleotide binding/exchange and NLR activation (206, 207).

**Figure 6.**
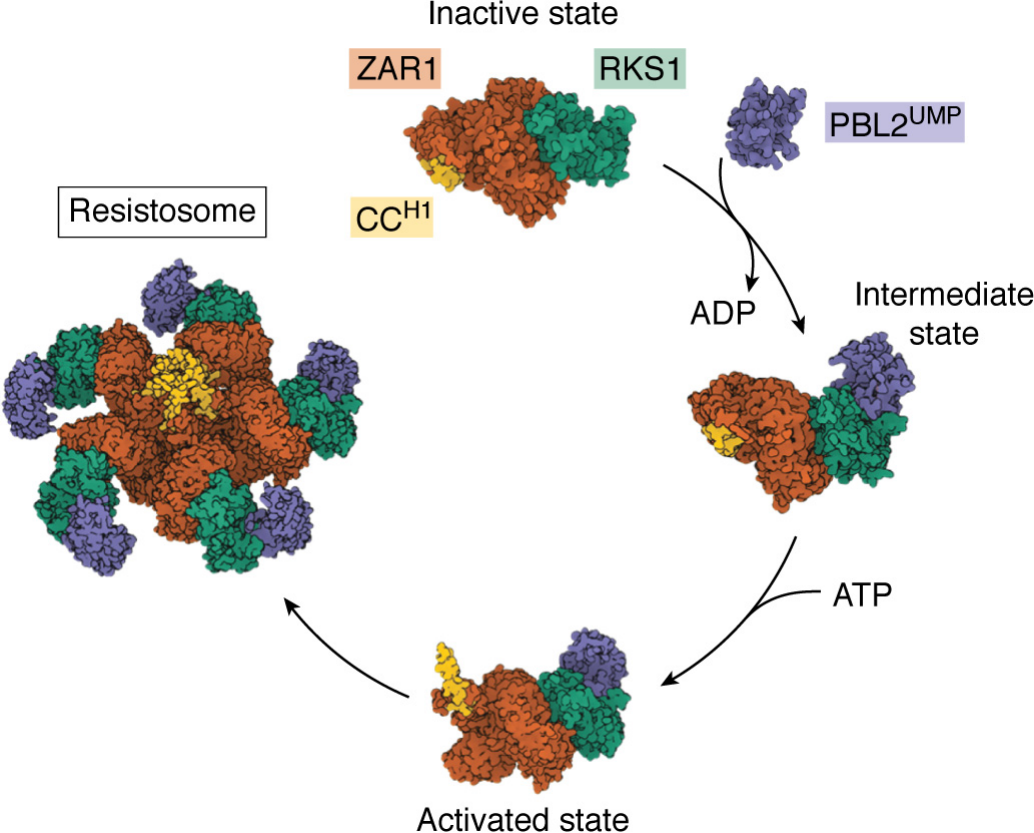
**The activation of the ZAR1 immune receptor.** ZAR1 (*orange*) is an *Arabidopsis* CC-NLR that forms complexes with pseudokinases, including ZED1 and RKS1 (*green*), to perceive effector activity (208). The ZAR1:RKS1 receptor complex guards the receptor-like cytoplasmic decoy kinase PBL2. Following uridylylation of PBL2 by the *Xanthomonas campestris* effector protein AvrAC, PBL2^UMP^ (*purple*) binds to RKS1, activating ZAR1. Activated ZAR1 is then able to oligomerize into a pentameric wheel with the CC domains each contributing their H1 helix (*yellow*) to form a funnel-like structure.

Conformational change and/or oligomerization of NLRs perturb the N-terminal CC or TIR domains to initiate immune responses. Whereas recent studies have begun to shed light on the molecular basis of how these domains trigger immunity, whether these reflect general principles applicable for all NLRs remains to be determined. For example, for CC domains, the structure of the *Arabidopsis* NLR ZAR1 (HopZ-activated resistance 1) revealed a mechanism whereby oligomerization results in a “funnel” of the N-terminal helices, which then associate with membranes and may perturb cellular integrity (14, 15) (Fig. 6). A sequence motif within the N-terminal helix of some NLR CC domains has been associated with ZAR1-like cell death immunity, known as the MADA motif (a consensus MADA*X*VSF*X*V*X*KL*XX*LL*XX*E*X* sequence conserved at the N termini of NRC (NLR required for cell death) family proteins) (209). This suggests that a subset of NLRs may function in a manner similar to ZAR1. However, how CC-NLRs that do not contain this motif function to initiate immunity has yet to be determined.

Many TIR domain structures from plant NLRs have been determined (16, 32, 35–37) and revealed multiple mechanisms of self-association to form scaffolds for protein-protein interactions that may be important for immune activation (210). Recently, the TIR domains of several NLRs have been shown to catalyze the hydrolysis of NAD^+^, suggesting a new mechanism for TIR-NLR activity (35, 211). How NAD^+^ hydrolysis functions in plant immunity is currently unknown. Recently, the structure of the *N. benthamiana* TIR-NLR Roq1 was determined, marking the first structure of a TIR-NLR resistosome (212). The Roq1 structure provides insight into the novel recognition of its cognate effector, XopQ, through interaction with a unique integrated-like domain deemed the post-LRR domain. Furthermore, it verifies the importance of specific TIR domain self-association interfaces, alluding to self-association resulting in the opening of the TIR domain active site for NAD^+^ binding and hydrolysis.

### NLRs function as singletons, pairs, and networks

To compensate for the lack of an adaptive immune system, plants have a diverse NLR repertoire, which has enabled functional specialization. This has resulted in the evolution of NLRs that function as singletons, in pairs, and as parts of interconnected networks (213–215) (Fig. 4*B*).

To date, several NLRs have been identified that appear to function autonomously, both sensing the presence of pathogens/pests and mounting a response. These are referred to as NLR singletons. Examples include NLRs of the mildew resistance locus A (MLA) in barley, *Arabidopsis* TIR-NLR RPP4 (recognition of *Peronospora parasitica* 4), and CC-NLR RPS2 (186, 216).

By contrast, other NLRs have specialized functions and can be broadly divided into two groups, sensors and helpers, and are generally referred to as NLR pairs (215). In these pairs, sensor NLRs perceive effectors, via the mechanisms discussed above, and helper NLRs are involved in converting effector perception into immune activation (181). NLR pairs can be genetically linked, often encoded at the same locus under the control of the same promoter. They are also always of the same class (CC-NLR or TIR-NLR). The best-characterized genetically linked sensor/helper paired NLRs are the *Arabidopsis* pair RRS1/RPS4, the rice pair Pik, and the rice pair Pia (also known as RGA5/RGA4). Intriguingly, each of the sensor NLRs of these pairs contains an integrated domain. General mechanisms for how paired NLRs function are based on models of suppression or receptor cooperation (217). The Pia and RRS1/RPS4 NLR pairs can be transiently expressed in tobacco leaves without clear cell death phenotypes. However, cell death phenotypes can be observed in tobacco leaves when RPS4 or the Pia helper NLRs are expressed without their cognate sensors or effectors. Co-expression of the RRS1 or Pia sensor NLRs suppresses the autoactive cell death phenotype of the helpers (218). Co-expression of the paired NLRs with their cognate effectors relieves this suppression, resulting in cell death. By contrast, expression of the Pik-2 helper NLR does not result in cell death, and co-expression of the Pik-1 sensor NLR and the cognate effector is required for cooperative cell death (11, 219).

However, a direct genetic link (head-to-head orientation or belonging to the same genetic loci) is not essential for NLR cooperation in immune activation, and some require complex NLR networks for function. The NLR “N-requirement gene 1” (NRG1), is required for the resistance to tobacco mosaic virus provided by the TIR-NLR, N (220). NRG1 is a member of the ADR1 (activated disease resistance 1) family of RPW8-NLRs, and since the discovery of NRG1, the RPW8-NLRs have been found to be important for the full function of many other CC-NLRs and TIR-NLRs (221, 222). Another NLR network has recently been uncovered in solanaceous plants. The NRCs are a phylogenetically distinct class of helper CC-NLRs consisting of functionally redundant paralogs (223). Sensor NLRs that require NRCs provide resistance to diverse pathogens and pests, including bacteria, oomycetes, nematodes, viruses, and insects (223). They display distinct specificities for different NRC helpers, with some sensors signaling through only one and others showing functional redundancy. Diversification of NLRs in the NRC network has allowed a varied arsenal of NLRs against a broad range of pathogens to have evolved.

Intriguingly, a new body of work has emerged, which has begun to uncover interplay between cell-surface and intracellular immunity (43, 44). These papers demonstrate that cell-surface immunity is required to potentiate intracellular immunity, enhancing NLR responses such as cell death. By contrast, NLR activity was shown to be important for cell-surface immunity receptor turnover, relieving attenuation of PRR signaling, and replenishing signaling components at the cell surface. These new findings open the door to further studies analyzing cross-network communications between cell-surface and intracellular immunity.

## Case study 3: The structure of ZAR1—the first plant resistosome

Recently, cryo-EM structures of ZAR1 were solved in inactive and active states. These are the first structures of full-length plant NLRs to be determined, and they represent a major advance in our understanding of NLR biology (14, 15). In the inactive state, the LRR domain in the ZAR1:RKS1 (resistance-related kinase 1) receptor complex makes autoinhibitory contacts with both the NB-ARC and CC domains, and a molecule of ADP is bound within the NB-ARC domain. PBL2^UMP^ binding to RKS1 induces a disorder-to-order transition of the RKS1 activation loop and a steric clash with the NB-ARC of ZAR1, which becomes displaced. This conformational change results in nucleotide exchange from bound ADP to ATP and stabilization of a structure primed for oligomerization with other activated RKS1:ZAR1 heterocomplexes. The pentamer that results from the oligomerization events is known as the “resistosome.” The conformational changes and oligomerization associated with PBL2^UMP^ binding promote unfolding of the ZAR1 CC domain, releasing the N-terminal helix (H1) from a four-helical bundle. The released H1 helix then associates with the H1 helices of neighboring activated ZAR1 molecules, resulting in the formation of a funnel-like structure with a striking hydrophobic surface. There is evidence that the ZAR1 CC domain funnel is required for membrane association and that this membrane association is linked to induction of cell death, potentially through ion efflux or membrane perturbation (14, 15, 224).

As this review was being finalized, the structure of the Roq1 TIR-NLR from *N. benthamiana* was determined by cryo-EM (212). This structure provides a significant advance in our understanding of plant NLR immunity as it represents the first structure of a TIR-NLR resistosome and, as such, should be considered alongside this ZAR1 case study.

### Next steps in understanding intracellular immunity

Despite recent advances, key questions on NLR function remain to be addressed. The ZAR1 and Roq1 structures have provided a wealth of information that has significantly expanded our understanding of plant NLR biology. However, it is as yet unclear how oligomerization of CC-NLRs into a resistosome mediates cell death. Furthermore, we lack structural information and evidence of resistosome formation for RPW8-NLRs. Even more perplexing is the role of TIR domain NADase activity and how it leads to the activation of RPW8-NLRs. Where we are beginning to generate a picture of the complex interactions between NLRs in plants, it is still unclear how one of the most primary interactions, effector detection, is mechanistically relayed from sensor NLRs to helper NLRs in pairs and networks. Each of these areas, among many others, requires further research to fully understand how NLRs provide resistance to pathogens/pests.

## Engineering plant immunity

Since their discovery, cell-surface and intracellular immune receptors have been targets of biotechnological approaches to improve disease resistance in plants. These approaches have included different scales, from transferring genes encoding plant receptors between species to specific amino acid mutations to modulate effector binding or receptor activity (52, 225). Engineering requires a holistic view, incorporating a range of methods to deliver both broad and robust resistance. Broad low-level resistance is regularly found in nature; however, due to monoculture reducing natural diversity, bespoke resistance to specific pathogens is often more desirable. Whereas the GMO debate remains a focus of public discussion and governmental policy decisions, engineering and editing crop genomes offers potential solutions to food insecurity.

### Engineering resistance by transferring genes

The transfer of traits conferring pathogen resistance is conceptually the most straightforward strategy to engineer disease immunity in plants. This method is used in classical plant breeding by selecting resistant phenotypes and crossing into desired cultivars. However, this approach is time-consuming and technically challenging (226). The recent development of new sequencing, phenotyping, and plant growth technologies has allowed researchers to overcome the limitations of this process (159, 227–229).

As plant cell-surface immune receptors tend to perceive common signatures of pathogens/pests and activate conserved signaling pathways in plants, they offer opportunities to transfer resistance between plant species. For example, the *Arabidopsis* EFR receptor is restricted to *Brassicaceae* species in nature but delivered novel resistance specificity against bacterial pathogens when it was transferred to tomato and rice (230–232). Similarly, transfer of the rice cell-surface receptor Xa21 to banana, sweet orange, or tomato increased resistance to *Xanthomonas* sp. (233–235). Further, an allelic FLS2 receptor from wild grape has been demonstrated to confer resistance to the crown-gall pathogen *Agrobacterium tumefaciens* when expressed in tobacco (236). Building on these advances, mining the diversity of cell-surface immune receptors with expanded recognition specificities from diverse plant species and their subsequent transfer to other plants, holds promise for engineering broad-spectrum disease resistance (230, 237, 238).

Recent advances in mining the genomes of wild plant species using new genomics technologies (228, 239–242) have allowed the rapid identification of candidate immune receptors for deployment in crops. Using such approaches, resistance to Asian soybean rust in soybean has been established by transferring an NLR from pigeon pea (243). Further, resistance has been shown against the potato late blight pathogen by introducing resistance genes from wild potato species (244, 245).

Plant intracellular NLR receptors are highly diverse (246–248) and often work together with other NLRs in pairs or networks. Therefore, NLRs frequently require a specific genetic background to provide effective disease resistance. As a consequence, the functional transfer of NLR receptors between species, or even cultivars of the same species, has proven challenging (249, 250).

### Bespoke engineering of NLR responses

Based on recent advances in our understanding of the mechanisms of NLR function, a number of new approaches are being explored to enable more effective engineering of NLRs to help deliver disease resistance in target plants.

### Domain exchange and mutagenesis

Domain exchange approaches between related NLRs have been explored for their potential to engineer disease resistance (251). Domain exchanges between the potato NLRs Rx and Gpa enabled the partial exchange of immune recognition from viruses to nematodes and vice versa (251). Autoactive and loss-of-function phenotypes were also observed in the chimeras and suggested that more subtle variations may have more potential.

High-throughput random mutagenesis of NLRs has been used to explore whether these receptors can be improved by enhancing their activation sensitivity or by expanding their recognition specificity. Following such approaches allowed expanded recognition of viruses by the NLR Rx (252, 253). This has been also applied to identify mutations that expanded the response of the potato NLR R3a and its tomato ortholog I-2 (254) to effectors from *Phytophthora* species (255). However, the translation of these expanded recognition phenotypes to disease resistance has remained limited (252–255). Recently, improved knowledge of how effectors, or effector activities, are directly perceived has inspired new methods of engineering.

### Decoy engineering

Understanding how NLRs that guard host targets are activated can allow engineering of recognition specificity. The *Arabidopsis* NLR RPS5 perceives cleavage of the decoy kinase PBS1 by the *P. syringae* effector AvrPphB at a specific recognition sequence (256). Mutation of the recognition site in PBS1 to cleavage sequences recognized by other translocated pathogen proteases, including a second *P. syringae* effector, AvrRpt2, and the Nla protease from tobacco etch virus, switched the RPS5 recognition specificity (257). It is of special note that the latter switched RPS5 perception from bacteria to viruses. Although this approach is limited to pathogens that translocate proteases into the host, the widespread conservation of this protease recognition systems in crop plants (258, 259) has recently allowed engineering of disease resistance in soybean (256).

### Integrated domains: New possibilities to engineer disease resistance

**Figure 7.**
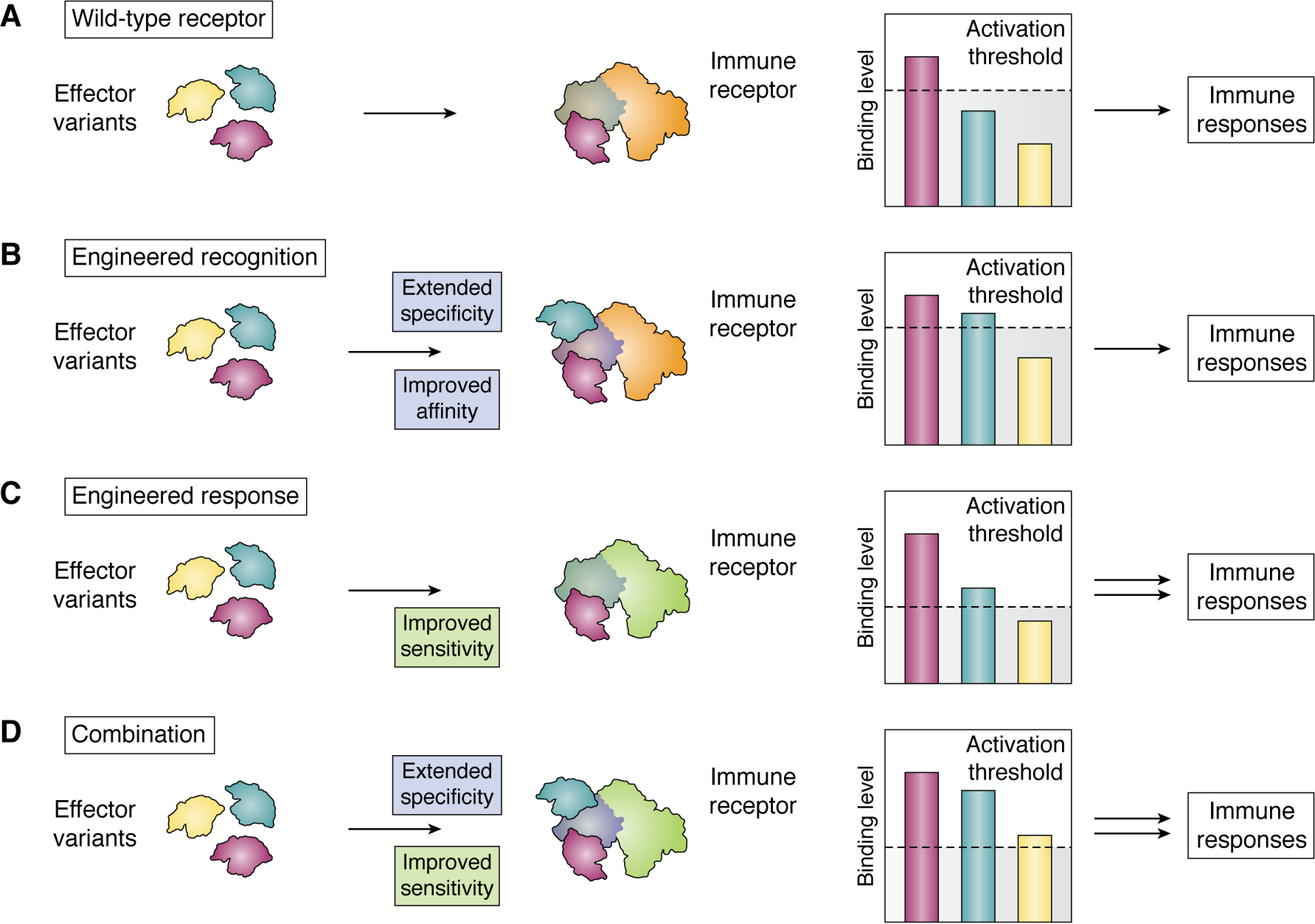
**Alternative strategies for immune receptor engineering.** *A*, plant immune receptors (*orange*/gray) bind natural variants of effector and ligands (*purple*/cyan/*yellow*) with different binding affinities (schematically depicted by the *height* of the *colored bars*). Only some binding events are of sufficient level to reach an activation threshold (represented by the *dashed line*), triggering immune responses. *B*, mutations in the receptor (*gray* to *blue*) can extend pathogen recognition by gaining or increasing binding to effectors and ligands, leading to immune responses to pathogens previously undetected. *C*, mutations in the immune receptors (*orange* to *green*) can lower the activation threshold, allowing for increased intensity of immune signaling. *D*, the combination of both mutations (*green*/*blue* receptor) that enhance recognition and immune responses can lead to wider and increased immune responses to effectors and ligands.

The discovery of integrated domains in plant NLRs opened new opportunities to understand and manipulate mechanisms of pathogen perception by intracellular immune receptors (177, 218, 260, 261) (Fig. 6). These domains have become a promising avenue for engineering disease resistance conferred by NLRs (205, 260, 261). As previously introduced, the allelic rice NLRs Pik perceive variants of the rice blast pathogen effector AVR-Pik by direct binding via an HMA domain (11, 29). Some natural effector variants are able to escape recognition by certain Pik NLR alleles, whereas other variants completely evade detection (29, 202, 262). Further, the binding of AVR-Pik effectors to the HMA is not in itself sufficient to activate immune signaling, and a threshold of binding needs to be reached to trigger immune responses (29) (Fig. 7*A*). An understanding of the biochemical and structural basis of different AVR-Pik/HMA interactions (29) has allowed the design of specific mutations that increase the binding affinity to effector alleles, expanding the recognition capability of the Pikp NLR (205) (Fig. 7*B*). This proof of concept demonstrated that NLR binding to effectors and the subsequent responses can be manipulated by rational design. Additional HMA domain engineering could now focus on extending perception of sequence-divergent rice blast effectors that also interact with HMAs, but at a different interface (30, 31, 190). Looking to the future, combining mutations in NLRs to both sensitize and lower the threshold to trigger immune responses, as discussed above (Fig. 7*C*), and directly increase binding affinity to effectors (Fig. 7*D*) is an exciting long-term goal for the field.

### Other approaches: Controlled expression of autoactive NLRs

A further possibility for engineering disease resistance is to manipulate expression of NLRs. For example, the discovery of a mechanism controlling defense responses at transcriptional and translational level allowed the design of a pathogen-responsive expression cassette (263). Placing an autoactive NLR under control of this cassette generated an NLR-mediated plant defense system that does not rely on effector recognition. This conferred broad-spectrum resistance without a fitness cost (264), a defense-yield trade-off that can occur when engineering immunity (265).

## Conclusion

Plant disease has shaped the natural and agricultural world. Crop losses due to disease and the emergence of resistant cultivars have been key events that have facilitated the way we breed and farm our food. Consequently, an understanding of the plant immune system is essential, as we attempt to develop new methods for disease control against a background of the climate emergency. Despite extensive studies, which we have reviewed here, further research is required to fully understand how the plant system works holistically to deliver disease resistance. Of the hundreds of cell-surface RLKs and RLPs identified, many of the biological functions and ligands of these receptors remain unknown. Furthermore, it is important to understand how plants distinguish the MAMPs of pathogens/pests from the MAMPs of the beneficial mutualist microbes. Determining the function of more of these cell-surface receptors will lead to new avenues for engineering resistance in crops. Similarly, advances in understanding NLR biology will help to better arm plants against pathogens and pests that evolve to circumvent cell-surface immunity. As we generate a better understanding of the complex interactions between plants and pathogens and pests, we can assemble the pieces to inform engineering of disease resistance, to produce more durable crops and help battle the food security problems of the future.
